# Association between social frailty and quality of life in older patients with chronic heart failure: sequential multiple mediating effects of family insufficiency and social networks

**DOI:** 10.3389/fmed.2025.1639935

**Published:** 2025-07-22

**Authors:** Junting Huang, Xiaobo Liu, Duolao Wang, Xiaorong Luan, Wanxia Yao

**Affiliations:** ^1^School of Nursing and Rehabilitation, Shandong University, Jinan, China; ^2^Nursing Department, Yan’an University Affiliated Hospital, Yan’an, Shaanxi, China; ^3^Liverpool School of Tropical Medicine, Liverpool, United Kingdom; ^4^Qilu Hospital of Shandong University, Jinan, China; ^5^School of Medicine, Xi'an Peihua University, Xi'an, China

**Keywords:** heart failure, social frailty, quality of life, family function, social networks

## Abstract

**Background and objectives:**

Older patients with chronic heart failure have severe somatic symptoms, which lead to high levels of social frailty and loss of quality of life. Understanding the demographic and disease factors of quality of life and its relationship with social frailty is beneficial to overall health for older patients with chronic heart failure. This study aims to explore the relationship between social frailty and quality of life in older patients with chronic heart failure and to verify whether family insufficiency and social networks moderate this relationship.

**Methods:**

A multi-centre cross-sectional study was conducted on 443 older patients with chronic heart failure from three tertiary hospitals in China. The study questionnaire included a general information questionnaire, the HALFT scale (social frailty), the Family APGAR Index (family insufficiency), the LSNS-6 (social networks), and the MLHFQ (quality of life). Hierarchical regression analysis was used to assess the factors influencing quality of life; the SPSS PROCESS Marco Plug-in was employed to conduct mediation analysis.

**Results:**

The results showed that age, the number of hospitalizations, and NYHA classification influenced the quality of life in older patients with chronic heart failure. Social frailty, family insufficiency, and social networks were related, and family insufficiency and social networks mediated the relationship between social frailty and quality of life, with mediating effect sizes of 25.87, 99.5 and 58.97%, respectively.

**Conclusion:**

This study shows that high levels of social frailty are associated with reduced quality of life in older patients with chronic heart failure. Decreasing family insufficiency and extending social networks help alleviate social frailty’s adverse effects on the quality of life in older patients with chronic heart failure.

## 1 Introduction

Chronic heart failure (CHF) is a progressive clinical syndrome resulting from myocardial damage, leading to impaired ventricular systolic or diastolic function (1). Globally, over 64.3 million individuals are affected by CHF, and its prevalence continues to rise due to population aging and improved survival rates (2). CHF is associated with frequent hospital readmissions, high mortality, and significant healthcare costs. More than 90% of CHF patients experience distressing somatic symptoms—such as dyspnoea, fatigue, oedema, and sleep disturbances—that severely compromise their quality of life (QOL) (3, 4).

According to the World Health Organization (WHO), health-related QOL reflects individuals’ perceptions of their position in life within their cultural and value contexts (5). In heart failure patients, QOL is a key clinical outcome encompassing physical, psychological, emotional, and social dimensions. Older CHF patients often report significantly lower QOL compared to individuals with hypertension, angina, or other chronic illnesses (6, 7). QOL is closely linked to re-hospitalizations and mortality rates. Moreover, severe somatic limitations and social withdrawal can lead to loneliness and deteriorating well-being (8). As an important component of social resources, leisure activities are as fundamental to QOL as health. Activities such as visiting friends, playing games, or running errands not only enhance social networks and emotional well-being but also play a key role in life satisfaction (9).

Social frailty (SF) refers to a persistent lack of access to essential social resources, such as emotional support, meaningful social roles, and financial security (10). Approximately two-thirds of older CHF patients experience SF, which is often associated with worse health outcomes, including poor disease management and reduced QOL (11). SF has also been linked to physical frailty, depression, and increased mortality (12–14). As the family remains a central pillar of support for older adults, its role in mitigating SF is critical. It is worth emphasizing that aging is a natural stage of life that brings with it a series of physical, psychological, and social changes. Taking measures to maintain or improve the QOL as we age is the key to active and healthy aging. Improving SF is one of the most recommended non-pharmacological strategies to improve the QOL in old age (15).

Family inadequacy (FI) is defined as the family’s diminished capacity to provide adequate care and support for older adults, often due to absence or insufficient resources (16). In dysfunctional families, poor care coordination can negatively affect medication adherence, diet, and daily routines, thereby lowering QOL (17). Family functionality has been identified as a protective factor against physical frailty and a determinant of QOL in older CHF patients (18). Previous studies have verified the correlation between family function and QOL of older adults, but there are currently few studies that use family insufficiency as a mediating variable to verify its role between SF and QOL in older CHF patients.

Social networks (SNs)—defined as reciprocal connections between individuals and society—play a key role in reducing loneliness and enhancing well-being (19). Strong SNs are associated with better cognitive status, lower risk of readmission, and improved QOL (20). These networks also help to alleviate the financial and emotional burden on families. This study is the first to use SNs and FI as mediating variables to verify their effects on the relationship between SF and QOL in older CHF patients.

To better understand the interplay between QOL, SF, FI, and SNs in older CHF patients, this study is guided by Maslow’s Hierarchy of Needs (21). This model categorizes human needs into five levels: physiological, safety, love/belonging, esteem, and self-actualization. CHF patients must simultaneously manage physical symptoms, ensure emotional and social support, and strive for psychological well-being. Based on this framework, we propose the following hypotheses:

*H1:* Social frailty independently affects QOL.

*H2:* SF, FI, SNs, and QOL are interrelated.

*H3:* FI and SNs mediate the relationship between SF and QOL.

**Figure 1:**
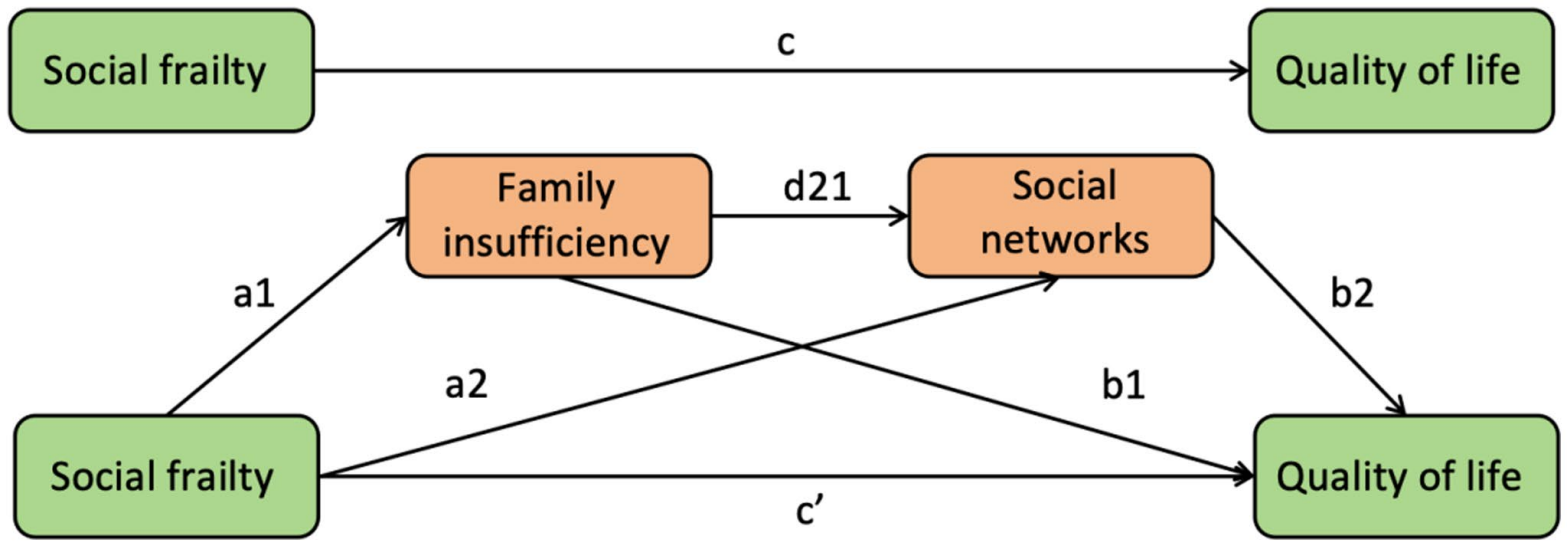
The chain-mediating model of family insufficiency and social networks between social frailty and quality of life. c = total effect of social frailty on quality of life. c’ = direct effect of social frailty on quality of life. a1*b1 = indirect effect of social frailty on quality of life via family insufficiency. a2*b2 = indirect effect of social frailty on quality of life via social networks. a1*d21*b1 = indirect effect of social frailty on quality of life via family insufficiency and social networks. Total effect (c) = direct effect (c’) + indirect effect (a1*b1) + indirect effect (a2*b2) + indirect effect (a1*d21*b1).

The conceptual framework is presented in Figure 1.

## 2 Materials and methods

### 2.1 Design

This is a multi-center cross-sectional study.

### 2.2 Participants

443 older patients with chronic heart failure were recruited from the Northeast (Shandong Province), Northwest (Shaanxi Province), and South (Guizhou Province) in China. The recruitment period was from October 2022 to June 2023. Participants aged ≥60 years with a confirmed CHF diagnosis according to established guidelines were eligible. Exclusion criteria included severe visual/hearing impairment, neurological disorders (e.g., dementia, stroke, epilepsy), psychiatric conditions (e.g., schizophrenia), and advanced terminal diseases.

**Figure 2:**
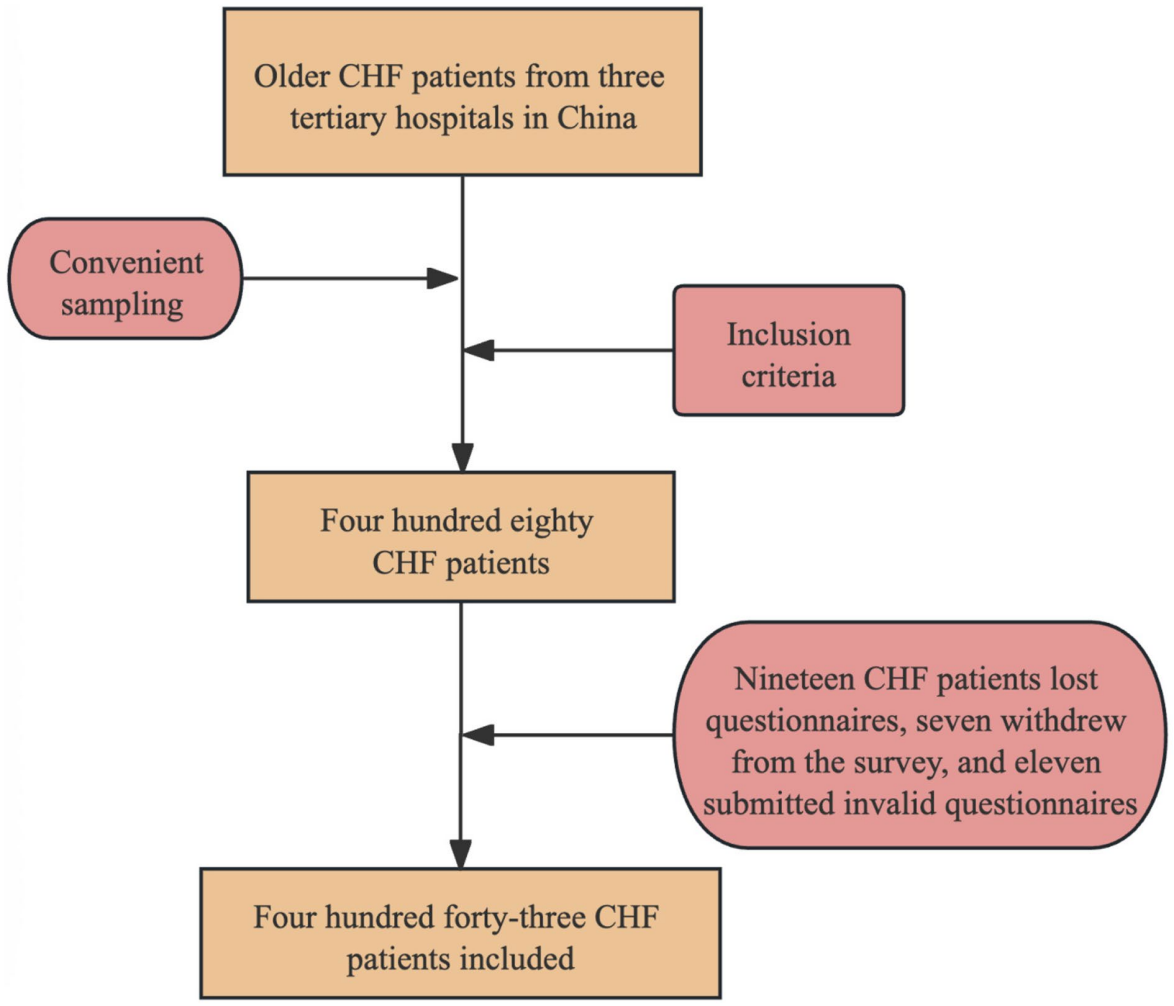
The flowchart process of participant recruitment.

Following the rule of thumb requiring 10–20 participants per variable, and assuming 14 variables, we estimated a required sample size of 140–280. Allowing for a 20% non-response or invalid questionnaire rate, the final target was 168–336 participants. Figure 2 shows the flowchart process of participant recruitment.

### 2.3 Ethics and procedures

This study strictly abides by the Declaration of Helsinki and has been approved by the School of Nursing and Rehabilitation Ethics Review Committee of Shandong University (approval no.: 2023-R-004; approval date: 3 February 2023).

Written informed consent was obtained from all participants. Data collection was conducted by a PhD student who had received systematic scientific research training and specialized questionnaire collection methods. Each interview and questionnaire completion took approximately 20–25 min. All participants were told they had the right to withdraw from the study without affecting subsequent treatment. All data were processed anonymously and destroyed after the end of the study.

### 2.4 Measurement instruments

Five questionnaires were used for data collection, namely the general information questionnaire, the Help, Participation, Loneliness, Financial, and Talk (HALFT) scale, the Family Adaptation, Partnership, Growth, Affect, and Resolve (APGAR) Index, the Lubben Social Network Scale-6 (LSNS-6), the Minnesota Living with Heart Failure Questionnaire (MLHFQ).

#### 2.4.1 General information questionnaire

This study used a general information questionnaire to collect covariates, including age, gender, marital status, residence, education level, monthly household income, number of hospitalizations, the New York Heart Association (NYHA) classes, and number of chronic diseases. All general information was obtained by a trained investigator reviewing the medical records. The coding of the category variables is affiliated by supplementary file.

#### 2.4.2 The help, participation, loneliness, financial, and talk (HALFT) scale

This study used the Help, Participation, Loneliness, Financial, and Talk (HALFT) scale to collect independent variable (social frailty). The HALFT scale consists of five dimensions: being unable to help others, limited social participation, loneliness, financial difficulties, and having no one to talk to. Five items: Item 1: Have you helped friends or family this past year? Item 2: Have you participated in social or leisure activities in the past year? Item 3: Have you felt lonely in the past week? Item 4: Was your income last year sufficient to cover your living expenses for one year? Item 5: Do you have someone to talk to every day? One point is calculated for ‘no’ answers to items 1, 2, 4, and 5, but for ‘yes’ answers to item 3. The total scores of the HALFT scale ranged from 0 to 5 points; the higher the scores were, the higher the SF level was. The Cronbach’s alpha coefficient of the HALFT scale is 0.736. The HALFT scale was developed by MA et al. specifically for Chinese older people and has been widely used in Chinese communities and clinical settings (22–24).

#### 2.4.3 The family adaptation, partnership, growth, affect, and resolve (APGAR) index

This study used the Family Adaptation, Partnership, Growth, Affect, and Resolve (APGAR) Index to collect mediator 1 (family insufficiency). The Family APGAR Index is a tool used to assess family functioning by examining five key dimensions: Adaptation, Partnership, Growth, Affection, and Resolve. It consists of five items, each corresponding to one of these dimensions, and each item is typically rated on a scale to determine the level of satisfaction within the family. Each item is scored by a three-point Likert scale ranging from 0 (not at all) to 2(always). The total score is 0–10 points, with the lower scores indicating the higher FI level. The Cronbach’s alpha coefficient of the Chinese version of the Family APGAR Index is 0.91 (25, 26).

#### 2.4.4 The Lubben social network scale-6 (LSNS-6)

This study used the Lubben Social Network Scale-6 (LSNS-6) to collect mediator 2 (social networks). The LSNS-6 consists of two dimensions: family network (3 items) and friends’ network (3 items). Six items: How many relatives do you see or hear from at least once a month? How many relatives do you feel at ease with that you can talk to about private matters? How many relatives do you feel close to such an extent that you could call on them for help? How many friends do you see or hear from at least once a month? How many friends do you feel at ease with that you can talk to about private matters? How many friends do you feel close to such an extent that you could call on them for help? Each item is scored by a six-point Likert scale (0 = none, 1 = one, 2 = two, 3 = three to four, 4 = five to eight, and 5 = nine and above). The total scores range from 0 to 30, with higher scores indicating more extensive social networks. The Cronbach’s alpha coefficient of the Chinese version of the LSNS-6 is 0.83 (27, 28).

#### 2.4.5 The Minnesota living with heart failure questionnaire (MLHFQ)

This study used the Minnesota Living with Heart Failure Questionnaire (MLHFQ) to collect the dependent variable (quality of life). The MLHFQ is a specialized tool for self-assessment of QOL in CHF patients. It consists of 21 items on a 0–5 Likert scale. The scale ranges from 0 to 105, representing the best to the worst QOL, more specially, higher MLHFQ scores indicate worse quality of life. The MLHFQ consists of two domains: the physical domain (8 items with a scale range of 0–40) and the emotional domain (5 items with a scale range of 0–25), and the remaining eight items are only used to calculate the total score. The Cronbach’s alpha coefficient of the Chinese version of the MLHFQ is 0.95 (29, 30).

### 2.5 Statistical analysis

IBM SPSS Statistics Version 26.0 (IBM, Armonk, NY, United States) was applied for data analysis. Category variables are described as frequencies and percentages. Numerical variables are expressed as means (16) and standard deviations (SD). Considering the lack of normal distribution of some variables based on Kolmogorov–Smirnov tests (*p*-value <0.05), Spearman correlation analysis opted to explore the relationships of variables. An independent sample t-test and ANOVA were used to perform a single-factor analysis of QOL. Stepwise Regression analysis was used for multiple-factor analysis of QOL. The value of R^2^ ranges between 0 and 1. The closer it is to 1, the better the model fits the data. The variance inflation factor (VIF) is less than 5, so there is no multicollinearity. In the regression analysis, covariates were added to Model 1, SF to Model 2, and FI and SN to Model 3. A threshold of 0.05 was established for statistical significance.

Multiple mediation was tested using PROCESS macro-Model 6 with 5,000 bootstrap samples to estimate indirect effects via family insufficiency (FI) and social networks (SNs). SF was set as X, FI was set as M1, SNs was set as M2, and QOL was set as Y. The indirect effects in this model include (1) through FI (a1b1), (2) through SNs (a2b2), and (3) through FI and SNs (a1d21b2). The sum of indirect effects = a1b1 + a2b2 + a1d21b2. If M1 and M2 are not present, the direct effect of X on Y is c’. The total effect of X on Y (16) is the direct effect + indirect effects: c = c’ + a1b1 + a2b2 + a1d21b2. All covariates were controlled in the mediation model. Bootstrap results were considered significant if the 95% confidence interval did not contain zeros.

## 3 Results

### 3.1 Participants’ characteristics

Four hundred eighty questionnaires were distributed for this study, of which 19 were lost, 7 participants were withdrawn, and 11 were invalid. Four hundred forty-three valid questionnaires were returned for a valid response rate of 93.81%. The main characteristics of the study participants are shown in Table 1. The mean age of the participants was 67.72 years (SD = 6.328), and the majority were female (63%). Approximately 11.3% of the participants were divorced, widowed, or single; 60.9% had an education level of primary school or below; 392 people were cohabiting with others, 51 were living alone; 169 people had two or more chronic diseases; 216 people were living in rural areas; the mean of the number of hospitalizations was 1.85(SD = 1.561); and 154 people had a monthly household income of 1,000 to 2,999 yuan. There were 96, 254, 68, and 25 people with NYHA classes I-IV, respectively. 124(28%), 176(39.7%), and 143(32.3%) people, respectively, came from Northwest, Northeast, and South China.

**Table 1:** Comparison of differences between quality of life in demographic and disease-related data (*n* = 443).

Variables	*n*(%)/mean (SD)	Quality of life		
		Mean (SD)	t/F/r value	*p* value
Age	67.72 (6.328)	40.83 (28.007)	−0.029	0.541
Gender			−0.215	0.830
Female	279 (63.0%)	40.61 (27.947)		
Male	164 (37.0%)	41.2 (28.19)		
Education level			7.522	<0.001
Primary school or illiterate	270 (60.90%)	37.17 (27.874)		
Secondary school	139 (31.40%)	44.77 (27.439)		
College or above	34 (7.70%)	53.76 (26.008)		
Marital status			0.512	0.609
Single	50 (11.30%)	42.74 (31.354)		
Married	393 (88.70%)	40.59 (27.586)		
Residence			−5.212	<0.001
Rural	227 (51.20%)	34.26 (26.975)		
Urban	216 (48.80%)	47.74 (27.461)		
Living situation			−0.82	0.413
Living alone	51 (11.50%)	37.8 (31.009)		
Living with others	392 (88.50%)	41.22 (27.611)		
Number of chronic diseases			0.126	0.90
None or one	274 (61.90%)	40.96 (27.491)		
Two or more	169 (38.10%)	40.62 (28.904)		
Number of hospitalizations	1.85 (1.561)	40.83 (28.007)	−0.111	0.019
Monthly household income			1.458	0.225
Less than 1,000 RMB	102 (23.0%)	45.1 (26.21)		
1,000 to 2,999 RMB	154 (34.80%)	41.07 (28.302)		
3,000 to 5,999 RMB	121 (27.30%)	37.28 (27.674)		
6,000 RMB or more	66 (14.90%)	40.17 (30.236)		
NYHA classification			3.109	0.026
Class I	96 (21.70%)	44.42 (26.117)		
Class II	254 (57.30%)	42.25 (30.418)		
Class III	68 (15.30%)	32.6 (16.828)		
Class IV	25 (5.60%)	34.96 (30.016)		
Distribution areas			1.091	0.355
Northwest China	124 (28.0%)	44.95 (27.33)		
Northeast China	176 (39.70%)	41.87 (28.96)		
South	143 (32.30%)	40.24 (26.15)		

### 3.2 Single-factor analysis of QOL

Independent sample t-test showed that residence (t = −5.212, *p* < 0.001) influences QoL. ANOVA results showed that NYHA classes (*F* = 3.109, *p* = 0.026) and education level (*F* = 7.522, *p* < 0.001) are influencing factors of QOL. Spearman correlation analysis showed that number of hospitalizations (r = −0.111, *p* = 0.019) is an influencing factor of QOL. Table 1 gives the details.

### 3.3 Correlation of SF, FI, SNs, and QOL

The mean level of SF is 2.37(SD = 1.461), the mean level of SNs is 15.51(SD = 6.138), the mean level of FI is 6.92(SD = 3.009), and the mean level of QOL is 40.83(SD = 28.007). Spearman correlation analysis indicated SF (r = 0.454, *p* < 0.01) was positively correlated with QOL. It also revealed that SNs (r = 0.520, *p* < 0.01) and FI (r = −0.685, *p* < 0.01) were negatively associated with QOL. Table 2 gives the details.

**Table 2:** Correlation analysis of social frailty, family insufficiency, social networks and quality of life (*n* = 443).

Variables	Mean (SD)	Quality of life	Social frailty	Social networks	Family insufficiency
Quality of life	40.83 (28.007)	1			
Social frailty	2.37 (1.461)	0.454**	1		
Social networks	15.51 (6.138)	−0.520**	−0.314**	1	
Family insufficiency	6.9 2(3.009)	−0.685**	−0.266**	0.467**	1

**p < 0.01.

### 3.4 Multiple-factor analysis of QOL

The multiple stepwise regression analysis results showed in model 1, adjusted R2 is 0.069, indicating that the number of hospitalizations, residence, and NYHA classes can predict QOL, with an explanation of 6.9%. In model 2, adjusted R2 is 0.27, indicating that SF can significantly expect QOL, with a net explanation of 20.1%. In model 3, R2 is 0.598, indicating that FI and SNs can predict QOL, with a net explanation of 32.8%. The main effect of SF on QOL is significantly reduced, suggesting that FI and SNs may mediate between SF and QOL. Table 3 gives the details.

**Table 3:** Regression analysis of quality of life (*n* = 443).

Variable	The first layer		The second layer		The third layer	
	Standardized beta	*p* value	Standardized beta	*p* value	Standardized beta	*p* value
Control variables						
Number of hospitalizations	−0.080	0.087	−0.082	0.047	−0.066	0.071
Class II	−0.040	0.485	−0.056	0.267	−0.02	0.654
Class III	−0.127	0.022	−0.159	0.001	−0.099	0.025
Class IV	−0.065	0.199	−0.076	0.087	−0.069	0.078
Urban	0.217	<0.001	0.152	0.003	0.119	0.009
Secondary school	0.002	0.968	0.007	0.882	0.012	0.777
College or above	0.062	0.233	0.048	0.295	0.035	0.396
Independent variables						
Social frailty			0.452	<0.001	0.219	<0.001
Mediators						
Family insufficiency					−0.477	<0.001
Social networks					−0.242	<0.001
Adjusted R^2^	0.069		0.27		0.598	
*F* value	5.675	<0.001	21.416	<0.001	66.686	<0.001

### 3.5 Mediation analysis of FI and SNs between SF and QOL

The bootstrap results indicated that the path standardized coefficient of SF on FI (path a1) was −0.2873 (95% CI: −0.7791, −0.4044), the path standardized coefficient of FI on QOL (path b1) was −0.4754 (95% CI: −5.0673, −3.7821), the path standardized coefficient of FI on SNs (path d21) was 0.3682 (95%CI: 0.5771, 0.9252), the path standardized coefficient of SF on SNs (path a2) was −0.2420 (−1.3744, −0.6593), the path standardized coefficient of SNs on QOL (path b2) was −0.2436 (−1.4371, −0.7861). The total effect (path c) and direct effect (path c’) of SF on QOL were 0.4529 (95% CI: 7.1090, 10.2560) and 0.2316 (95% CI: 3.1750, 5.7049), respectively. The total indirect effect of SF on QOL (path a1*b1 + path a1*d21*b1 + path a2*b2) is 0.2213 (95% CI: 0.1635, 0.2774). The indirect impact of sequential multiple mediating effects of FI and SNs is 95.55%, calculated by (path a1*b1 + path a1*d21*b1 + path a2*b2)/(path c’), and is a partial mediation. The mediating effect size of FI is 58.97%, calculated by (path a1*b1)/(path c’). And the mediating effect size of SNs is 25.87%, calculated by (path a2*b2)/(path c’). Table 4 gives the details.

**Table 4:** Mediating analysis of social frailty, family insufficiency, social networks, and quality of life (*n* = 443).

Model path	Standardized coefficient	BootSE	BootLLCI	BootULCI
Path a1				
Social frailty→ Family insufficiency	−0.2873	0.0953	−0.7791	−0.4044
Path b1				
Family insufficiency → Quality of life	−0.4754	0.3269	−5.0673	−3.7821
Path d21				
Family insufficiency → Social networks	0.3682	0.0885	0.5771	0.9252
Path a2				
Social frailty → Social networks	−0.2420	0.1819	−1.3744	−0.6593
Path b2				
Social networks → Quality of life	−0.2436	0.1656	−1.4371	−0.7861
Total effect (c)				
Social frailty → Quality of life	0.4529	0.8006	7.1090	10.2560
Direct effect (c’)				
Social frailty → Quality of life	0.2316	0.6435	3.1750	5.7049
Indirect effect1 (a1*b1)	0.1366	0.0228	0.0913	0.1820
Indirect effect2 (a2*b2)	0.0590	0.0143	0.0333	0.0881
Indirect effect3 (a1*d21*b2)	0.0258	0.0067	0.0143	0.0402
Total indirect effect (a1*d21*b2 + a1*b1 + a2*b2)	0.2213	0.0293	0.1635	0.2774

## 4 Discussion

The study examined the association between social frailty (SF) and quality of life (QOL) for older patients with chronic heart failure (CHF) coming from Northwest, Northeast, and South China. It also explored the effects of demographic and disease factors and family insufficiency (FI) and social networks (SNs) on QOL. In addition, it is the first study to verify the chain mediating role of FI and SNs between SF and QOL. Furthermore, the findings confirm that SF directly affects QOL and indirectly through FI and SNs, which suggests that reducing SF is crucial in enhancing QOL in older CHF patients.

We found residence, New York Heart Association (NYHA) classes and number of hospitalizations were influential factors for the QOL of older CHF patients. The QOL of older CHF patients living in rural areas was higher than that in urban areas, which is consistent with Chantakeeree’s study (31). Considering that China’s ‘basic medical insurance system’ and ‘medical assistance policy for major illnesses’ have gradually covered rural areas, these policies positively reduce the financial burden and improve the QOL of rural old adults (32, 33). In addition, older adults in rural areas often engage in more physical activities (e.g., farming, walking, etc.), which can help to improve cardiovascular health and slow down the symptoms of CHF. The NYHA classes of CHF are divided into four classes according to the patient’s ability to exercise and the severity of the symptoms: Class I means no symptoms, and Class IV means unable to perform any physical activities (1). The QOL in Classes II and III was higher than in classes I and IV. Probably due to patients in NYHA class I lack knowledge about CHF and had inadequate CHF-related care. Patients in NYHA class IV could not perform basic activities of daily living due to the severity of their symptoms, which contributed to the decrease in QOL. In addition, with the personalized treatment of older CHF patients with NYHA classes II and III, including medication modification, physical training and psychological support, the functional status and QOL of older patients have improved (34, 35). In the study, the QOL status of older CHF patients with more hospitalizations was better but inconsistent with Luiso’s study (36). For older patients with CHF, hospitalization is usually a sign of acute exacerbation or complications. However, during hospitalization, patients can receive strict fluid management, dietary control and medication, reducing the discomfort of CHF symptoms such as dyspnoea and oedema and improving the QOL (37, 38). In addition, considering the dietary differences between northern and southern China and the higher level of development of community hospitals in the south, these may also be important reasons for the increase in the number of hospitalizations among older CHF patients.

SF in older CHF patients was strongly associated with QOL in this study. Specifically, older CHF patients who lacked social participation, social contact, financial support, and a sense of loneliness had lower levels of QOL, consistent with the results of Odaci’s study (39). Physical symptoms often accompany older CHF patients and have a higher prevalence of SF due to the prolonged course of the disease, high hospitalization rate, and recurrent acute exacerbations. A study found that approximately 40% of older CHF patients exhibited SF and physical frailty (11). A cohort study has revealed about 35% of older CHF patients were diagnosed with SF (40), consistent with a high prevalence of SF in Europe (14). SF reflect the general inadequacy of social support and participation in activities in older CHF patients. Studies have shown that SF usually leads to reduced social and physical activity in patients, affecting their physical functioning (41, 42). Coupled with the fact that CHF already impacts physical functioning, if SF accompanies it, the decline in the patient’s physical functioning may be even more pronounced, leading to a loss of quality of life. In addition, SF patients usually have more severe depression and anxiety, and these psychological problems are also strongly associated with the chronic course of CHF and QOL.

There is a significant negative correlation between FI and QOL for older CHF patients in the study, meaning the higher the FI level, the worse the patient’s QOL. Family plays a vital role in treating and rehabilitating older CHF patients. However, the family functionality of many older CHF patients is constrained by various factors, manifested as insufficient family support, family role disorder, and excessive care pressure, leading to the loss of QOL of older CHF patients. In this study, the FI of older CHF patients was moderate, consistent with Shabani’s findings (43). A study about the comparison of QOL scores of patients with atrial fibrillation across different FI statuses revealed that patients in the low FI group had higher scores on overall QOL, fewer symptoms (e.g., chest tightness, palpitations, dizziness, etc.), fewer treatment worries, and less limited daily activities compared to those in the high FI group (44). Another study on the relationship between FI and QOL in patients with type 2 diabetes mellitus also found that a higher level of FI leads to lower QOL levels, which is consistent with our study (45). In addition, considering the influence of traditional Chinese culture, such as ‘raising children to prevent old age’ and ‘having many children is a blessing’, the family plays an indispensable role in older patients with chronic diseases.

In older CHF patients, the SNs are negatively correlated with CHF-related symptoms. In other words, the stronger the SNs, the fewer the CHF-related symptoms, and the better the QOL. Enhanced SNs can help older CHF patients capture disease-related knowledge, adjust unhealthy life behaviors, and reduce the risk of disease exacerbation, increasing QOL (19, 46, 47). In China, the SNs formed based on blood and geographic ties are characterized by a “differential pattern”. Older people’s daily lives and social activities are deeply embedded in their SNs. Compared with previous studies, we found that the difference in QOL between older people from different regions was not significant. Although previous studies have shown that the level of QOL of older people in the south is higher than that in the north based on the multi-level of recreational activities and the development of medical facilities, this is inconsistent with the results of this study. In addition, with the acceleration of population mobility, the traditional model of living with children in old age has changed, and the SNs are more vital to their physical and mental health. Studies revealed that older patients receiving active SN support have more social participation, reducing loneliness and increasing patients’ happiness (48–50). At the same time, Zhang et al. suggested that there may be a bidirectional moderating relationship between SNs and physical activity in chronic disease patients, demonstrating SNs’ importance in older adults (51). In the pessimistic prediction of numerous chronic diseases through SNs in older adults, social participation is gradually enriched with the expansion of SNs, and physical activities are relatively increased, improving the QOL (52).

This study verified that FI and SN have partial mediating effects between SF and QOL, respectively, and that FI and SN also play a chain mediating role. A strong bond is formed between chronic disease patients and their families. This support helps to build and enhance self-confidence, cope with illness, and manage emotions. In addition, as part of the social support system, families play a key role in adopting and maintaining health-promoting behaviors (43). They can, therefore, enhance patients’ QOL by providing positive social support, including emotional and informational support. Social support can strengthen social networks, fulfil the need for social connectedness, and ultimately reduce individuals’ feelings of loneliness and improve their QOL. Anna et al. compared foreign-born and native-born individuals in Sweden and found that inadequate social resources generally meant lower levels of social support, which further led to mental health inequalities between native-born and immigrant Swedish (53). Family relationships can expand the social network of older people, thereby providing more opportunities to obtain social support. Compared to previous studies, we further found good family relationships and social networks are conducive to promoting physical exercise among patients, improving the flexibility of bones, muscles, and joints; furthermore, improving the functional level of the cardiovascular, respiratory, nervous, and motor systems, and helping to increase immunity. In addition, good physical function enables older people to participate in social activities, thereby reducing the sense of loneliness caused by social isolation, reducing SF, and enhancing QOL.

## 5 Limitations

This study has some limitations. First, this study is a cross-sectional study that cannot determine the causal relationship between variables. It is recommended that future longitudinal studies be conducted to investigate further the chain-mediating effect of FI and SNs on SF and QOL. Second, China faces the impact of the ‘new elderly care model’, which runs counter to the traditional ‘filial piety’ culture. However, this study did not include cultural variables, which limits the generalizability of the results. In addition, we collected data through a self-reported questionnaire, which may be subject to recall bias. However, the measurement tools in this study are all reliable and valid. Finally, we used convenience sampling, which may limit the generalizability to some extent. However, this study adopted a multicenter study and the sample was representative. It is recommended to use complex stratified sampling in the future.

## 6 Relevance to clinical practice and policy

Healthy aging is a major challenge that all countries globally are facing (54, 55). How to effectively help older individuals live healthily and safely is an important task in promoting health equity. Cardiovascular disease is a major disease that seriously endangers the physical, mental and social health of older individuals, and chronic heart failure, as the terminal stage of cardiovascular disease, urgently needs to be paid attention to. This study aimed to explore the mediating roles of family insufficiency and social networks between social frailty and quality of life in older patients with chronic heart failure. Our findings provide scientific evidence for clinical healthcare workers to provide efficient and precise drug and non-drug interventions for older patients. Furthermore, we also provide policymakers with new perspectives on how to promote healthy aging.

## 7 Conclusion

We found residence, New York Heart Association (NYHA) classes and number of hospitalizations were influential factors for the QOL of older CHF patients. Furthermore, we first verified the sequential mediating effects of FI and SNs between SF and QOL in older CHF patients. Medical and nursing professionals should expand the family cohesion and social networks of older patients, such as conducting family communication activities and broadening the social circles of older patients. In addition, policymakers should also pay attention to the social aspects of the health of older individuals and promote the comprehensive development of healthy aging.

## Data Availability

The original contributions presented in the study are included in the article/supplementary material, further inquiries can be directed to the corresponding authors.

## References

[1] Abovich A Matasic DS Cardoso R Ndumele CE Blumenthal RS Blankstein R. The AHA/ACC/HFSA 2022 heart failure guidelines: changing the focus to heart failure prevention. Am J Prev Cardiol. (2023) 15:100527. doi: 10.1016/j.ajpc.2023.100527, PMID: 37637197 PMC10457686

[2] Hollenberg SM Stevenson LW Ahmad T Bozkurt B Butler J Davis LL. 2024 ACC expert consensus decision pathway on clinical assessment, management, and trajectory of patients hospitalized with heart failure focused update: a report of the American College of Cardiology solution set oversight committee. J Am Coll Cardiol. (2024) 84:1241–67. 39127954 10.1016/j.jacc.2024.06.002

[3] Hu SS. Heart failure in China: epidemiology and current management. J Geriatr Cardiol. (2024) 21:631–41. doi: 10.26599/1671-5411.2024.06.008, PMID: 38973826 PMC11224652

[4] Aïdoud A Gana W Poitau F Debacq C Leroy V Nkodo JA. High prevalence of geriatric conditions among older adults with cardiovascular disease. J Am Heart Assoc. (2023) 12:e026850. doi: 10.1161/JAHA.122.026850, PMID: 36628962 PMC9939057

[5] The World Health Organization quality of life assessment (WHOQOL): position paper from the World Health Organization. Soc Sci Med. (1995) 41:1403–9. doi: 10.1016/0277-9536(95)00112-K, PMID: 8560308

[6] Lawson CA Benson L Squire I Zaccardi F Ali M Hand S. Changing health related quality of life and outcomes in heart failure by age, sex and subtype. EClinicalMedicine. (2023) 64:102217. doi: 10.1016/j.eclinm.2023.102217, PMID: 37745020 PMC10514432

[7] Savarese G Becher PM Lund LH Seferovic P Rosano GMC Coats AJS. Global burden of heart failure: a comprehensive and updated review of epidemiology. Cardiovasc Res. (2023) 118:3272–87. doi: 10.1093/cvr/cvac013, PMID: 35150240

[8] Li Vigni M Ghiadoni L Pugliese N. The impact of heart failure on quality of life: a mixed method study. Eur Heart J Suppl. (2024) 26:ii84–5. doi: 10.1093/eurheartjsupp/suae036.203

[9] Parra-Rizo MA Vásquez-Gómez J Álvarez C Diaz-Martínez X Troncoso C Leiva-Ordoñez AM. Predictors of the level of physical activity in physically active older people. Behav Sci (Basel). (2022) 12. doi: 10.3390/bs12090331, PMID: 36135135 PMC9495331

[10] MaLSunFTangZ. Social frailty is associated with physical functioning, cognition, and depression, and predicts mortality. J Nutr Health Aging. (2018) 22:989–95. doi: 10.1007/s12603-018-1054-0, PMID: 30272104

[11] Jujo K Kagiyama N Saito K Kamiya K Saito H Ogasahara Y. Impact of social frailty in hospitalized elderly patients with heart failure: a FRAGILE-HF registry subanalysis. J Am Heart Assoc. (2021) 10:e019954. doi: 10.1161/JAHA.120.019954, PMID: 34472374 PMC8649263

[12] Wang Z Ruan H Li L Song N He S. Association of changes in frailty status with the risk of all-cause mortality and cardiovascular death in older people: results from the Chinese longitudinal healthy longevity survey (CLHLS). BMC Geriatr. (2024) 24:96. doi: 10.1186/s12877-024-04682-2, PMID: 38267867 PMC10809745

[13] Doi T Tsutsumimoto K Makino K Nakakubo S Sakimoto F Matsuda S. Combined social frailty and life-space activities associated with risk of disability: a prospective cohort study. J Frailty Aging. (2024) 13:184–8. doi: 10.14283/jfa.2024.17, PMID: 38616376 PMC12275790

[14] Yu S Wang J Zeng L Yang P Tang P Su S. The prevalence of social frailty among older adults: a systematic review and meta-analysis. Geriatr Nurs. (2023) 49:101–8. doi: 10.1016/j.gerinurse.2022.11.009, PMID: 36470103

[15] Patel R Gallagher JE. Healthy ageing and oral health: priority, policy and public health. BDJ Open. (2024) 10:79. doi: 10.1038/s41405-024-00262-z, PMID: 39379352 PMC11461822

[16] Abdi S Spann A Borilovic J de Witte L Hawley M. Understanding the care and support needs of older people: a scoping review and categorisation using the WHO international classification of functioning, disability and health framework (ICF). BMC Geriatr. (2019) 19:195. doi: 10.1186/s12877-019-1189-9, PMID: 31331279 PMC6647108

[17] Chokkanathan S Mohanty J. Health, family strains, dependency, and life satisfaction of older adults. Arch Gerontol Geriatr. (2017) 71:129–35. doi: 10.1016/j.archger.2017.04.001, PMID: 28432920

[18] de Oliveira Tavares ML Pimenta AM García-Vivar C Beinner MA Montenegro LC. Determinants of quality of life decrease in family caregivers of care-dependent patients: a longitudinal study. Qual Life Res. (2025) 34:365–75. doi: 10.1007/s11136-024-03814-w, PMID: 39425868 PMC11865120

[19] Stephens C Bakhshandeh Bavarsad M. Social network type contributes to purpose in life among older people, mediated by social support. Eur J Ageing. (2024) 21:5. doi: 10.1007/s10433-024-00799-w, PMID: 38231456 PMC10794679

[20] Wang Y-L Chen Y-J Liu C-C. The relationship between social media usage and loneliness among younger and older adults: the moderating effect of shyness. BMC Psychology. (2024) 12:343. doi: 10.1186/s40359-024-01727-4, PMID: 38863021 PMC11167928

[21] Sirgy MJ. A quality-of-life theory derived from Maslow's developmental perspective. Am J Econ Sociol. (1986) 45:329–42. doi: 10.1111/j.1536-7150.1986.tb02394.x

[22] Li Z Gu J Li P Hu J Wang S Wang P. The relationship between social frailty and loneliness in community-dwelling older adults: a cross-sectional study. BMC Geriatr. (2024) 24:73. doi: 10.1186/s12877-024-04666-2, PMID: 38238657 PMC10797967

[23] Huang C Sirikul W Buawangpong N. Social frailty in community-dwelling older adults: a scoping review. BMC Geriatr. (2025) 25:329. doi: 10.1186/s12877-025-05971-0, PMID: 40360975 PMC12070721

[24] Zhang X-M Cao S Gao M Xiao S Xie X Wu X. The prevalence of social frailty among older adults: a systematic review and Meta-analysis. J Am Med Dir Assoc. (2023) 24:29–37.e9. doi: 10.1016/j.jamda.2022.10.007, PMID: 36402197

[25] Nan H Ni MY Lee PH Tam WW Lam TH Leung GM. Psychometric evaluation of the Chinese version of the subjective happiness scale: evidence from the Hong Kong FAMILY cohort. Int J Behav Med. (2014) 21:646–52. doi: 10.1007/s12529-014-9389-3, PMID: 24515396 PMC4107280

[26] Cheng Y Zhang L Wang F Zhang P Ye B Liang Y. The effects of family structure and function on mental health during China's transition: a cross-sectional analysis. BMC Fam Pract. (2017) 18:59. doi: 10.1186/s12875-017-0630-4, PMID: 28476107 PMC5420133

[27] Chang Q Sha F Chan CH Yip PSF. Validation of an abbreviated version of the Lubben social network scale ("LSNS-6") and its associations with suicidality among older adults in China. PLoS One. (2018) 13:e0201612. doi: 10.1371/journal.pone.0201612, PMID: 30071067 PMC6072030

[28] Yang K Liu Y Yin X Wu S Wu Q Wang L. A survey of social network status and its related factors for older adults with type 2 diabetes in Beijing, China. Nurs Open. (2022) 9:1005–14. doi: 10.1002/nop2.1138, PMID: 34850591 PMC8859026

[29] Cong J Zhu Y Du J Lin L He Y Zhang Q. Mapping the Minnesota living with heart failure questionnaire (MLHFQ) to SF-6Dv2 in Chinese patients with heart failure. Health Qual Life Outcomes. (2022) 20:98. doi: 10.1186/s12955-022-02004-x, PMID: 35725609 PMC9208129

[30] Zhu Y Cong J Lin L Du J Long L He Y. Minimum clinically important differences in the Minnesota living with heart failure questionnaire: from a study of heart failure patients treated with integrated Chinese and Western medicine. Front Cardiovasc Med. (2023) 10 2023. doi: 10.3389/fcvm.2023.1242216, PMID: 38089764 PMC10711109

[31] Chantakeeree C Sormunen M Estola M Jullamate P Turunen H. Factors affecting quality of life among older adults with hypertension in urban and rural areas in Thailand: a cross-sectional study. Int J Aging Hum Dev. (2022) 95:222–44. doi: 10.1177/00914150211050880, PMID: 34931879 PMC9316351

[32] Wang X Chen X Li L Zhou D. The impacts of basic medical insurance for urban–rural residents on the perception of social equity in China. Cost Effectiv Res Alloc. (2024) 22:57. doi: 10.1186/s12962-024-00565-w, PMID: 39097696 PMC11298076

[33] Wu W Long S Cerda AA Garcia LY Jakovljevic M. Population ageing and sustainability of healthcare financing in China. Cost Effect Res Alloc. (2023) 21:97. doi: 10.1186/s12962-023-00505-0, PMID: 38115117 PMC10729482

[34] Gallagher A Lucas R Cowie M. 39 does NYHA class predict health-related quality of life? Heart. (2018) 104:A37–7.

[35] Gallagher AM Lucas R Cowie MR. Assessing health-related quality of life in heart failure patients attending an outpatient clinic: a pragmatic approach. ESC Heart Fail. (2019) 6:3–9. doi: 10.1002/ehf2.12363, PMID: 30311454 PMC6352889

[36] Luiso D Herrero-Torrus M Badosa N Roqueta C Ruiz-Bustillo S Belarte-Tornero LC. Quality of life in older patients after a heart failure hospitalization: results from the SENECOR study. J Clin Med. (2022) 11. doi: 10.3390/jcm11113035, PMID: 35683423 PMC9181457

[37] Veskovic J Cvetkovic M Tahirovic E Zdravkovic M Apostolovic S Kosevic D. Depression, anxiety, and quality of life as predictors of rehospitalization in patients with chronic heart failure. BMC Cardiovasc Disord. (2023) 23:525. doi: 10.1186/s12872-023-03500-8, PMID: 37891464 PMC10612261

[38] Guo Y Sun J Hu S Nicholas S Wang J. Hospitalization costs and financial burden on families with children with depression: a cross-section study in Shandong Province, China. Int J Environ Res Public Health. (2019) 16. doi: 10.3390/ijerph16193526, PMID: 31547207 PMC6801864

[39] Odaci Comertoglu E Ozturk Y Hafizoglu M Kahyaoglu Z Cavusoglu C Balci C. The effect of social frailty on mental health and quality of life in older people: a cross-sectional study. Eur Geriatr Med. (2024) 15:453–61. doi: 10.1007/s41999-024-00931-0, PMID: 38332388

[40] Ragusa FS Veronese N Smith L Koyanagi A Dominguez LJ Barbagallo M. Social frailty increases the risk of all-cause mortality: a longitudinal analysis of the English longitudinal study of ageing. Exp Gerontol. (2022) 167:111901. doi: 10.1016/j.exger.2022.111901, PMID: 35870753

[41] Nagai K Tamaki K Kusunoki H Wada Y Tsuji S Itoh M. Physical frailty predicts the development of social frailty: a prospective cohort study. BMC Geriatr. (2020) 20:403. doi: 10.1186/s12877-020-01814-2, PMID: 33054731 PMC7557012

[42] Qi X Li J. The relationship between social frailty and depressive symptoms in the elderly: a scoping review. Int J Environ Res Public Health. (2022) 19. doi: 10.3390/ijerph192416683, PMID: 36554564 PMC9779347

[43] Shabani M Amini-Tehrani M Sadri M Taheri-Kharameh Z Khaljinia Z Poorolajal J. Family function, social support and quality of life in community-dwelling older adults: the moderating role of gender. Curr Psychol. (2024) 43:690–7. doi: 10.1007/s12144-023-04297-7

[44] Tian M Kang J Huan X Yin J Zhang Z. Correlation between family function and quality of life in patients with atrial fibrillation. Zhong Nan Da Xue Xue Bao Yi Xue Ban. (2023) 48:1234–42. doi: 10.11817/j.issn.1672-7347.2023.220551, PMID: 37875364 PMC10930848

[45] Zan H Meng Z Li J Zhang X Liu T. Factors associated with quality of life among elderly patients with type 2 diabetes mellitus: the role of family caregivers. BMC Public Health. (2024) 24:539. doi: 10.1186/s12889-024-17917-z, PMID: 38383369 PMC10880260

[46] Marzo RR Jun Chen HW Ahmad A Thew HZ Choy JS Ng CH. The evolving role of social media in enhancing quality of life: a global perspective across 10 countries. Arch Public Health. (2024) 82:28. doi: 10.1186/s13690-023-01222-z, PMID: 38449000 PMC10918911

[47] Bincy K Logaraj M Anantharaman VV. Social network and its effect on selected dimension of health and quality of life among community dwelling urban and rural geriatric population in India. Clin Epidemiol Global Health. (2022) 16:101083. doi: 10.1016/j.cegh.2022.101083

[48] Aliverdi F Farajidana H Tourzani ZM Salehi L Qorbani M Mohamadi F. Social networks and internet emotional relationships on mental health and quality of life in students: structural equation modelling. BMC Psychiatry. (2022) 22:451. doi: 10.1186/s12888-022-04097-6, PMID: 35790935 PMC9255442

[49] Majmudar IK Mihalopoulos C Abimanyi-Ochom J Mohebbi M Engel L. The association between loneliness with health service use and quality of life among informal carers in Australia. Soc Sci Med. (2024) 348:116821. doi: 10.1016/j.socscimed.2024.116821, PMID: 38569284

[50] Briere J Wang SH Khanam UA Lawson J Goodridge D. Quality of life and well-being during the COVID-19 pandemic: associations with loneliness and social isolation in a cross-sectional, online survey of 2,207 community-dwelling older Canadians. BMC Geriatr. (2023) 23:615. doi: 10.1186/s12877-023-04350-x, PMID: 37777717 PMC10542692

[51] Smith BJ Lim MH Manera KE Phongsavan P Owen KB. Bidirectional relationships between loneliness, social isolation, and physical inactivity in the household, income and labour dynamics in Australia cohort study. Ann Behav Med. (2024) 58:619–27. doi: 10.1093/abm/kaae043, PMID: 39066664 PMC11305128

[52] Schmidt T Christiansen LB Schipperijn J Cerin E. Social network characteristics as correlates and moderators of older adults’ quality of life—the SHARE study. Eur J Pub Health. (2021) 31:541–7. doi: 10.1093/eurpub/ckab001, PMID: 33547475 PMC8530164

[53] Brydsten A Rostila M Dunlavy A. Social integration and mental health—a decomposition approach to mental health inequalities between the foreign-born and native-born in Sweden. Int J Equity Health. (2019) 18:48. doi: 10.1186/s12939-019-0950-1, PMID: 30944004 PMC6889340

[54] Mendorf S Schönenberg A Heimrich KG Prell T. Prospective associations between hand grip strength and subsequent depressive symptoms in men and women aged 50 years and older: insights from the survey of health, aging, and retirement in Europe. Front Med (Lausanne). (2023) 10:1260371. doi: 10.3389/fmed.2023.1260371, PMID: 37780562 PMC10536140

[55] Gao J Xu J Chen Y Wang Y Ye B Fu H. Development and validation of a multidimensional population-based healthy aging scale: results from the China health and retirement longitudinal study. Front Med (Lausanne). (2022) 9:853759. doi: 10.3389/fmed.2022.853759, PMID: 35237637 PMC8882972

